# Association between hypothyroidism and risk of chronic kidney disease: evidence from a systematic review and meta-analysis

**DOI:** 10.3389/fendo.2026.1704228

**Published:** 2026-02-11

**Authors:** Wei-Kun Zhuang, Xin-Yu Hu, Yun-Jia An, De-Liang Liu, Hui-Lin Li

**Affiliations:** 1 Shenzhen Traditional Chinese Medicine Hospital Affiliated to Nanjing University of Chinese Medicine, Shenzhen, Guangdong, China; 2 Classic Department of Traditional Chinese Medicine, Meitan County Hospital of Integrated Chinese and Western Medicine, Zunyi, Guizhou, China; 3 The Second Affiliated Hospital, School of Medicine, The Chinese University of Hong Kong, Shenzhen & Longgang District People’s Hospital of Shenzhen, Shenzhen, Guangdong, China; 4 Department of Traditional Chinese Medicine, Meitan Hospital Affiliated to Zunyi Medical University, Zunyi, Guizhou, China; 5 Department of Endocrinology, Shenzhen Traditional Chinese Medicine Hospital, Shenzhen, Guangdong, China

**Keywords:** chronic kidney disease, hypothyroidism, meta-analysis, risk factors, systematic review

## Abstract

**Background:**

Chronic kidney disease (CKD) has become a significant issue in global public health, with its prevalence showing a persistent upward trend. Traditional risk factors cannot fully explain the persistent increase in incidence rates. Hypothyroidism, a common endocrine disorder, is considered a potential non-traditional risk factor for CKD, but the evidence remains inconsistent. This study aims to systematically evaluate the association between hypothyroidism and CKD risk.

**Methods:**

Search PubMed, Embase, Web of Science, and Cochrane Library databases for relevant literature up to September 1, 2025. Included observational studies reporting the association between overt or subclinical hypothyroidism and CKD risk, providing odds ratios (OR) or hazard ratios (HR) with 95% confidence intervals (CIs). Quality assessment was conducted using the Newcastle–Ottawa Scale (NOS) and the Agency for Healthcare Research and Quality (AHRQ) quality assessment tool. Calculate pooled risk estimates using random-effects models, and conduct subgroup analyses, sensitivity analyses, and publication bias analyses.

**Results:**

A total of 13 publications reporting 15 studies (11 cross-sectional and 4 cohort studies) involving approximately 5,101,102 participants were included. The pooled analysis of cross-sectional studies demonstrated that hypothyroidism was significantly associated with CKD (OR = 1.66, 95% CI: 1.43–1.94; *I*² = 78.6%). Subgroup analyses revealed consistent associations across different thyroid disease types, regions, diagnostic methods, and study quality group. Pooled results from cohort studies also demonstrated that hypothyroidism significantly increased CKD risk (HR = 1.21, 95% CI 1.08-1.36; *I*² = 50.08%). Sensitivity analysis results remained stable. Publication bias was present in cross-sectional studies, but the association remained significant after using the trim-and-fill method (adjusted OR = 1.29, 95% CI: 1.06–1.57).

**Conclusion:**

This systematic review and meta-analysis suggests that hypothyroidism is associated with an increased risk of CKD. Future well-designed prospective and interventional studies are needed to clarify temporal and causal relationships and to elucidate the underlying mechanisms between hypothyroidism and renal dysfunction.

**Systematic Review Registration:**

https://www.crd.york.ac.uk/prospero/, identifier CRD420251143834.

## Introduction

Chronic kidney disease (CKD), defined as persistent structural or functional kidney abnormalities lasting at least three months and leading to health complications (1), has become a growing global public health challenge. In 2017, an estimated 697.5 million people worldwide were affected across all stages of CKD (95% uncertainty interval: 649.2–752.0 million), corresponding to a prevalence of 9.1% (8.5%–9.8%), with an increasing trend (2). This high prevalence underscores the urgent need for more effective prevention strategies. Previous studies have identified key risk factors for CKD, including age, hypertension, diabetes, obesity, proteinuria, dyslipidemia, and salt intake (3). However, these known factors cannot fully explain the persistent rise in CKD incidence. Increasing evidence suggests that non-traditional systemic factors, particularly endocrine and metabolic dysregulation, may play a significant role in the onset and progression of CKD (4, 5). Given the close physiological interactions between thyroid hormones and renal hemodynamics, glomerular filtration, and tubular function, thyroid dysfunction is increasingly recognized as a potential factor contributing to the onset and progression of CKD.

Hypothyroidism is a common thyroid dysfunction. Overt hypothyroidism is characterized by thyroid-stimulating hormone (TSH) levels above the reference range and free thyroxine (FT4) levels below the reference range, whereas subclinical hypothyroidism presents with elevated TSH but normal FT4 levels (6). Recent large-scale population-based data indicate that the prevalence of hypothyroidism has shown a continuous upward trend over the past decade. A study by Wyne et al. (7) found that the overall prevalence of hypothyroidism in the United States increased from approximately 9.5% in 2012 to 11.7% in 2019, with subclinical hypothyroidism showing particularly marked growth. Hypothyroidism is more common in women and the elderly, and its systemic effects extend far beyond metabolic regulation. Given the high prevalence of hypothyroidism and its extensive physiological impact, increasing attention is being directed toward the potential effects of hypothyroidism on dysfunction in organ systems beyond the thyroid itself, particularly those systems highly sensitive to hemodynamic and hormonal regulation.

Among these organ systems outside the thyroid, the kidneys are particularly susceptible to changes in thyroid hormone status. Thyroid hormones can influence renal function by indirectly affecting cardiac output and renal blood flow changes, or by directly influencing glomerular filtration rate, tubular secretion and reabsorption, and electrolyte balance (8, 9). Recent evidence indicates that individuals with hypothyroidism exhibit a higher incidence of CKD compared to the general population (8, 10, 11). Moreover, compared with CKD patients with normal thyroid function, those with hypothyroidism exhibit poorer renal outcomes and a higher risk of progression to end-stage renal disease and mortality (10, 12, 13). Nevertheless, the results across different studies are not entirely consistent. A large cohort study involving 4,152,830 patients found that individuals with hypothyroidism had a 25% higher risk of developing CKD compared to those with normal thyroid function (14). Additionally, a previous meta-analysis of diabetic populations reported a significant association between hypothyroidism and CKD risk (OR 1.22; 95% CI 1.10–1.36), further supporting the potential link between thyroid dysfunction and renal outcomes despite its findings being limited to diabetic patients (15). However, a cohort study involving 11,872 patients found that the association between hypothyroidism and CKD risk was not statistically significant (HR = 1.01, 95% CI 0.83–1.21) (16). These inconsistent findings highlight the need for a comprehensive evaluation of the relationship between hypothyroidism and CKD risk.

Previous meta-analyses have only examined the relationship between subclinical hypothyroidism and the risk of CKD, or have been limited to specific populations (diabetic individuals) (15), and the number of included studies was limited (17). To address this research gap, we conducted a systematic review and meta-analysis to comprehensively evaluate the association between hypothyroidism (including overt and subclinical forms) and CKD risk. By integrating data from diverse populations, this study aims to provide more generalizable evidence regarding the association between hypothyroidism and CKD risk.

## Methods

### Registration of review protocol

This study was reported according to PRISMA (Preferred Reporting Items for Systematic Reviews and Meta-Analyses) and was prospectively registered in PROSPERO (registration number: CRD420251143834).

### Search strategy

Two researchers (X.Y.H. and Y.J.A.) independently retrieved publications from the Web of Science, PubMed, Cochrane Library, and EMBASE databases from their inception through September 1, 2025. The search terms for this study combined Medical Subject Headings (MeSH) with free-text terms, including: “hypothyroidism” or “subclinical hypothyroidism” or “thyroid dysfunction” or “thyroid insufficiency” and “chronic kidney disease” or “renal insufficiency” or “chronic renal insufficiency” or “kidney insufficiency”. We considered only studies written in English and based on human subjects. To identify potentially eligible articles, we also searched and read the full references from original studies. For detailed search strategies, details are provided in **Supplementary Table 1**.

### Study selection

The inclusion criteria for this meta-analysis are as follows (1): Observational studies (cross-sectional, cohort, or case-control studies) examining the association between hypothyroidism and the risk of CKD (2). Complete data were reported, including odds ratio (OR) or hazard ratio (HR), along with 95% confidence intervals (CIs). (3) Published in English. (4) The research is based on human subjects. (5) The diagnosis of hypothyroidism is based on measurements of TSH and FT4 concentrations or on self-reported medical history, along with the use of levothyroxine replacement therapy.

The exclusion criteria for this meta-analysis are as follows: (1) conference abstracts, case reports, reviews, practice guidelines, commentaries, or editorials; (2) Studies examining the association between serum TSH levels or thyroid hormone levels and CKD risk. (3) Studies involving only individuals with normal thyroid function; (4) Studies were not included if they did not report a dichotomous CKD outcome based on eGFR <60 mL/min/1.73 m² and only analyzed continuous changes in eGFR.

### CKD definition

CKD was predefined as the primary outcome. CKD was defined as including an estimated glomerular filtration rate (eGFR) <60 mL/min/1.73 m², with or without renal injury markers such as proteinuria or albuminuria. Studies were eligible if CKD was determined through laboratory measurements (eGFR derived from serum creatinine), regardless of the specific equation used (MDRD or CKD-EPI) (18, 19). Studies defining CKD solely based on self-report without biochemical confirmation were excluded.

### Data extraction and quality assessment

Two researchers (X.Y.H. and Y.J.A.) independently extracted and cross-checked data from studies ultimately included in the meta-analysis. Any disagreements between researchers regarding the inclusion of eligible studies were resolved through consensus and consultation with a third researcher (D.L.L.). We extracted the following data from each study: publication year, country, study design, sample size, mean age, male proportion, follow-up duration, diagnostic methods for hypothyroidism and CKD, type of thyroid disease, and matched and confounding factors adjusted for in multivariate regression analysis.

Two researchers conducted quality assessments of cohort studies using the Newcastle-Ottawa Scale (NOS) and of cross-sectional studies using the Agency for Healthcare Research and Quality (AHRQ) criteria (20). Studies scoring below 5 points are rated as low quality, those scoring 5–7 points are rated as moderate quality, and studies scoring 8 points or above were rated as high quality. All disagreements were resolved through mutual agreement and discussion with another author.

### Statistical analyses

We used OR from cross-sectional studies and HR from longitudinal studies as effect sizes (ES), each with 95% confidence intervals. When studies reported OR/HR values with varying degrees of covariate adjustment, we selected the OR/HR that most fully reflected the potential confounding variables.

The statistical heterogeneity of each study was assessed using the *I²* statistic. A random-effects model was applied when heterogeneity was substantial (*I²* ≥ 50%), whereas a fixed-effect model was used when heterogeneity was low to moderate (*I²* < 50%). The *I²* statistic is classified into low heterogeneity (<25%), moderate heterogeneity (25%-75%), and high heterogeneity(>75%) (21). To explore potential sources of heterogeneity among studies and assess the robustness of findings, subgroup analyses were conducted based on region, type of thyroid disease, thyroid diagnostic method, and risk of bias. To assess the robustness of the results, we employed a leave-one-out analysis to examine potential over-influence from individual studies (22). Potential publication bias was analyzed using funnel plots and the Egger’s test when ≥10 studies were available (23), and further explored using the Duval and Tweedie nonparametric trim-and-fill methods (24).

All statistical tests in this study were two-tailed, with *P* < 0.05 considered statistically significant. Statistical analysis was performed using R software (version 4.3.2; packages ‘meta’ and ‘metafor’).

## Results

### Literature search

**Figure 1:**
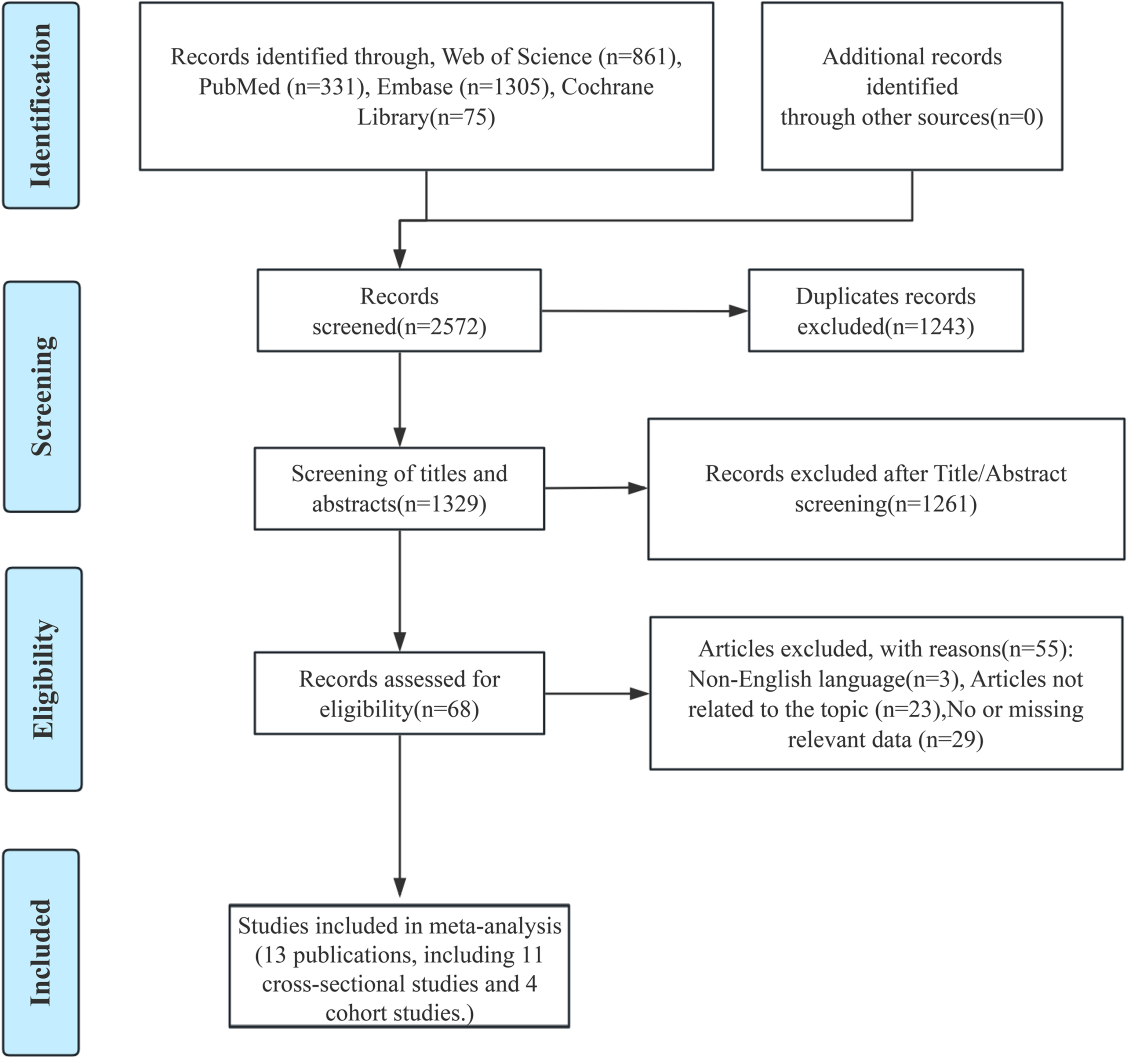
Flow chart of study selection of hypothyroidism in relation to CKD.

**Figure 1** presents the flowchart of the literature screening process. A systematic search of studies published before September 1, 2025 yielded a total of 2,572 results. After initial screening, 1,243 duplicate records were excluded. Additionally, based on title and abstract screening, 1,261 articles were excluded for irrelevance to the study topic. The remaining 68 studies underwent full-text review. A total of 13 publications reporting 15 studies (11 cross-sectional and 4 cohort studies), with some publications reporting multiple studies. These studies reported on the relationship between hypothyroidism and CKD risk.

### Study characteristics

A total of 13 publications reporting 15 studies published between 2011 and 2024 were included in this meta-analysis, comprising 11 cross-sectional studies and 4 cohort studies (10, 13, 14, 16, 25–33), with a combined sample size of 5,101,102 participants. In terms of the study population, 8 studies were conducted on people of all ages, and 2 studies were conducted on people with diabetes. The relationship between subclinical hypothyroidism and CKD was discussed in 4 studies, while the relationship between hypothyroidism and CKD was discussed in 10 studies, and 1 study did not specify whether it was hypothyroidism or subclinical hypothyroidism. In all 15 included studies, CKD was defined using an eGFR threshold <60 mL/min/1.73 m², with 5 studies additionally incorporating criteria for proteinuria or albuminuria. Among cross-sectional studies, 10 studies determined CKD based on a single eGFR measurement, while 1 study used multiple measurements. Among the cohort studies, 4 studies used repeated eGFR measurements or predefined longitudinal algorithms during follow-up to determine CKD or event-driven CKD. All studies based CKD determination on laboratory measurements, none relied solely on diagnostic codes or self-reported CKD. In terms of research regions, there were 3 studies in the United States, 3 studies each in China and Japan, and 1 study each in South Korea, Australia, South Africa, Brazil, and Norway. Although the potential confounding factors adjusted for (e.g., age, gender, ethnicity, smoking) varied across studies, all included studies used adjusted effect values. **Table 1** shows the main characteristics of the included studies.

**Table 1:** Characteristics of the studies included in the quantitative and qualitative review.

First Author, year	Country	Study design	Sample size	Mean age(years)	Male sex(%)	Median follow-up time(years)	CKD diagnosis	Diagnosis of hypothyroidism	hypothyroidism	Covariate adjustments	NOS score	AHRQ score
Huang, 2020 (10)	United States	Cross-sectional	378101	67( ± 9)	43%	/	eGFR <60mL/min/1.73 m2	an elevated level of TSH (>4.00mIU/L) and/or receipt of thyroid hormone replacement (levothyroxine sodium, thyroid, thyroid strong, bovine thyroid, pork thyroid, liothyronine sodium, and liotrix)	hypothyroidism	age, sex, race, hypertension and diabetes	/	8
Shimizu, 2022 (26)	Japan	Cross-sectional	1724	40~74	37%	/	eGFR <60mL/min/1.73 m2	TSH > 4.01 μIU/mL	subclinical hypothyroidism	sex, age, systolic blood pressure (SBP), body mass index (BMI), drinking status, smoking status, triglycerides (TG), high-density lipoprotein cholesterol (HDLc), glycohemoglobin (HbA1c)	/	7
Jia, 2015 (31)	China	Cross-sectional	933	61.8 ± 12.1	47%	/	GFR < 60 ml/min/1.73 m^2^ or a UACR > 2.5 mg/mmol in men and > 3.5 mg/mmol in women	Patients with normal levels of free triiodothyronine and free thyroxine, but an increased level of TSH (reference range: 0.35–5.00 mU/l)	subclinical hypothyroidism	age, gender, diabetes duration, hypertension, smoking and drinking status, BMI and HbA1c	/	7
Gopinath, 2013 (32)	Australia	Cross-sectional	1571	≥49	40.80%	/	eGFR <60mL/min/1.73 m2	TSH > 4.0 mIU/L and FT4 < 11.5 pmol/L	hypothyroidism	age, sex, receipt of pension payment, smoking, body mass index, hypertension, and diabetes	/	9
Kim, 2023	Korea	Cross-sectional	3257	44.10 ± 0.28	54.10%	/	eGFR < 60 mL/min/1.73m^2^ and/or ACR ≥ 30 mg/g	TSH levels > 6.68 mIU/L and normal fT4 levels	subclinical hypothyroidism	Age, sex, household income, education, smoking, alcohol consumption, walking activity, abdominal obesity, hypertension, low high-density lipoprotein cholesterol, elevated triglycerides, hyperglycemia, free thyroxine and thyroid-peroxidase antibody	/	8
Schultheiss 2017 (16)	United States	Cross-sectional	12109	57.4 ± 5.7	43.50%	/	eGFR <60mL/min/1.73 m2	TSH concentrations above 5.1 mIU/L (5.4 for white persons and 4.2 for black persons) and FT4 below 10.9 pmol/L (for race-specific analyses: white persons, <11.2 pmol/L; black persons, <10.6 pmol/L)	hypothyroidism	age, gender, race, serum albumin, BMI, hs-CRP, smoking status, systolic blood pressure, diabetes, LDL and HDL cholesterol, triglycerides, hypertension and medication use for cholesterol and DM	/	9
Johnson, 2020 (27)	South Africa	Cross-sectional	310	62(54-71)	47.10%	/	eGFR < 60 mL/min/1.73m^2^ and/or ACR ≥ 30 mg/g	T4 less than the normal range (7.6–16.1 pmol/l) and TSH > 4 mIU/l	hypothyroidism	/	/	5
Peixoto De Miranda, 2017 (29)	Brazil	Cross-sectional	13193	51(45-58)	48.20%	/	eGFR <60mL/min/1.73 m2	TSH level above 4.0 mIU/l, normal FT 4 level, no thyroid medication used	subclinical hypothyroidism	sex, race, hypertension, diabetes, dyslipidemia, smoking status, cardiovascular disease, BMI, and albumin: creatinine ratio	/	9
Åsvold 2011 (33)	Norway	Cross-sectional	12302	57(41-98)	33.10%	/	eGFR <60mL/min/1.73 m2	TSH >4.0 mU/l and FT4 <8.0 pmol/l	hypothyroidism	sex, age and smoking	/	9
Toda, 2019 (28)	Japan	Cross-sectional	16390	54 ± 11.2	67.80%	/	eGFR <60mL/min/1.73 m2	TSH>4.26 mU/L	hypothyroidism	age, sex, obesity, hypertension, dyslipidemia, hyperuricemia, hyperglycemia, proteinuria, and hematuria	/	8
Chang, 2018 (18)	China	Cross-sectional	74356	41.7 ± 13.4	50.20%	/	eGFR <60mL/min/1.73 m2	TSH > 5 mIU/L	hypothyroidism	age, sex, mean blood pressure, fasting glucose, BMI, total cholesterol, triglycerides, uric acids, physical inactivity, cigarette smoking, alcohol consumption, low income level, low education level and proteinuria	/	8
Schultheiss, 2017 (16)	United States	Cohort	11872	57.4 ± 5.7	43.50%	19.6	eGFR <60mL/min/1.73 m2	TSH concentrations above 5.1 mIU/L (5.4 for white persons and 4.2 for black persons) and FT4 below 10.9 pmol/L (for race-specific analyses: white persons, <11.2 pmol/L; black persons, <10.6 pmol/L)	hypothyroidism	age, gender, race, serum albumin, BMI, hs-CRP, smoking status, systolic blood pressure, diabetes, LDL and HDL cholesterol, triglycerides, hypertension and medication use for cholesterol and DM	9	/
You, 2024 (14)	United States	Cohort	4152830	55 ± 16	41%	3.8	eGFR <60mL/min/1.73 m2	TSH level >5.0 mIU/L	hypothyroidism	age, sex, race/ethnicity, CCI score, diabetes, coronary artery disease (CAD), congestive heart failure (CHF), atrial fibrillation, hyperlipidemia and hypertension	8	/
Chuang, 2016 (30)	China	Cohort	74356	75.8 ± 6.4	51.90%	3	eGFR <60mL/min/1.73 m2	TSH level >10 mIU/L	hypothyroidism	Sex, age, hypertension, diabetes mellitus, dyslipidemia (low HDL-C, high LDL-C, hypertriglyceridemia), hyperuricemia, abnormal liver function (GPT >42 U/L), anemia, obesity, smoking and alcohol drinking	8	/
Toda, 2019 (28)	Japan	Cohort	7609	52.8 ± 10.1	68.70%	3	eGFR <60mL/min/1.73 m2	TSH>4.26 mU/L	/	age, sex, obesity, hypertension, dyslipidemia, hyperuricemia, hyperglycemia, proteinuria, and hematuria	8	/

### Quality assessment

The NOS scale and AHRQ scale are used for quality assessment in cohort studies and cross-sectional studies, respectively. The average quality score for the 15 studies was 8 points, with 12 studies scoring 8 points or higher. The scores for each study are shown in **Table 1**, and the scoring criteria for each study are shown in **Supplementary Table 2**-**Supplementary Table 3**.

### Cross-sectional study of hypothyroidism and CKD risk

Eleven cross-sectional studies analyzed the relationship between hypothyroidism and the risk of CKD. These studies included a total of 514,246 participants, with eight studies scoring 8 points or higher on the AHRQ score and three studies scoring below 8 points, indicating overall moderate to high quality with a generally low risk of bias.

**Figure 2:**
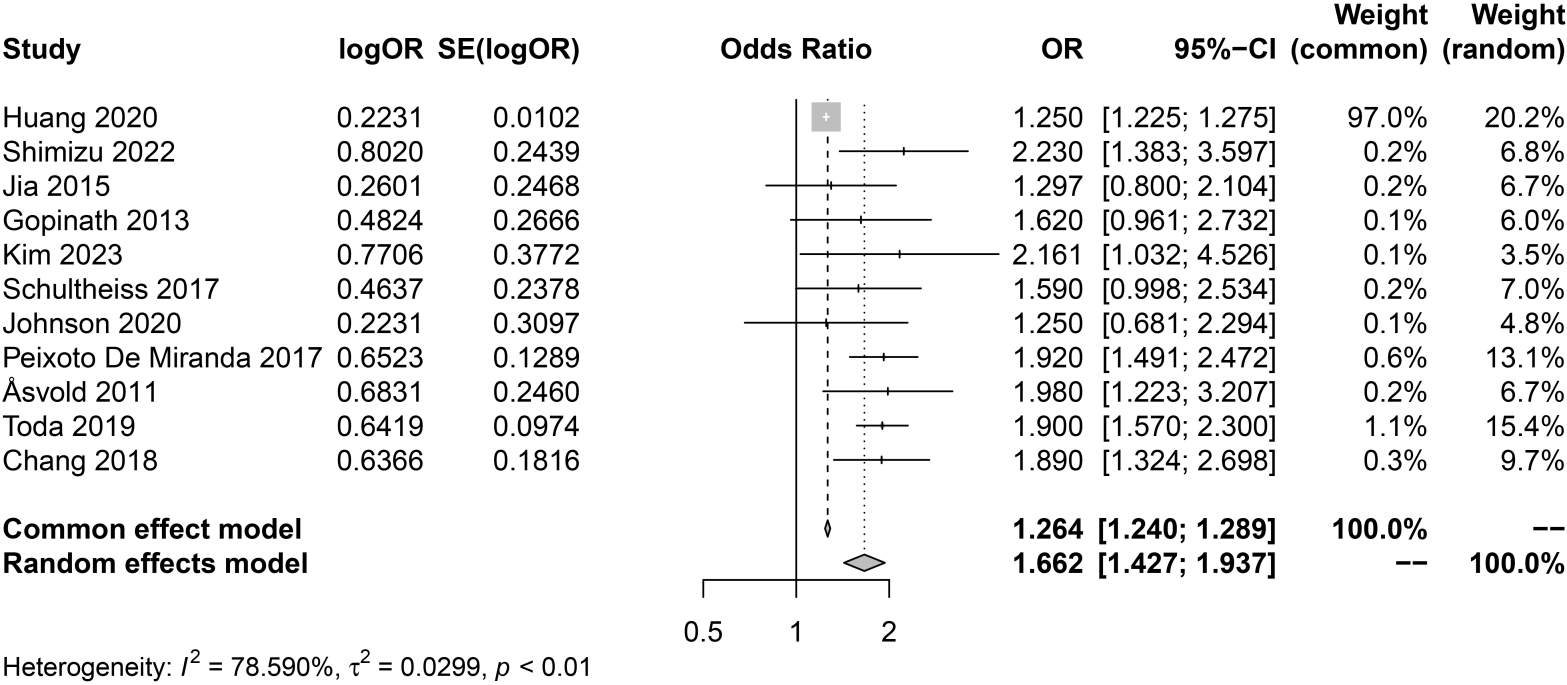
Forest plot of cross-sectional studies on the association between hypothyroidism and CKD risk.

The meta-analysis of cross-sectional studies on hypothyroidism and the risk of CKD is shown in **Figure 2**. Due to the high degree of heterogeneity (*I*^2^ = 78.59%), we applied a random-effects model for analysis. The combined analysis of 11 cross-sectional studies revealed a significant positive association between hypothyroidism and the risk of CKD (pooled random-effects OR = 1.66, 95% CI 1.43-1.94; *I*^2^ = 78.59%, *P*<0.01). Since we used fully adjusted estimates, the pooled random effects OR values were independent of risk factors such as age, gender, ethnicity, smoking, and diabetes.

### Subgroup analysis of cross-sectional studies

To further explore potential sources of heterogeneity and validate the stability of results, we conducted several subgroup analyses. Subgroup analyses were conducted based on cross-sectional studies due to the small number of studies included in the cohort study and all of them were overt hypothyroidism population. As shown in **Supplementary Figure 1**, a stratified comparison was performed according to the type of hypothyroidism. A total of 7 studies included hypothyroid populations and 4 studies included subclinical hypothyroid populations. Hypothyroidism was significantly positively associated with CKD risk in both groups. The hypothyroidism group showed a lower random-effects OR and higher heterogeneity (n=7 studies, OR = 1.59, 95% CI 1.32-1.92; *I*^2^ = 79.02%, *P*<0.01), suggesting that the heterogeneity in the meta-analysis may originate from within the hypothyroidism group.

Subgroup analysis of the study by region showed that the association between hypothyroidism and CKD risk was consistent across different regions. There were 5 studies from the Asian region and 6 from the non-Asian region. As shown in **Supplementary Figure 2**, the random-effects OR values were lower in non-Asian regions and showed significant heterogeneity (n=6 studies, OR = 1.54, 95% CI 1.25-1.89; *I*^2^ = 69.46%, *P*<0.01).

Subgroup analysis based on the diagnostic methods for hypothyroidism showed that there was still a significant positive correlation between hypothyroidism and CKD risk in both the TSH-based diagnosis group and the TSH and FT4-based diagnosis group. A total of 3 studies diagnosed on the basis of TSH and 8 studies diagnosed on the basis of TSH+FT4. As shown in **Supplementary Figure 3**, the group diagnosed using TSH showed a significant association, but there was extremely high heterogeneity (n=3 studies, OR = 1.66, 95% CI 1.18-2.35; *I*^2^ = 91.60%, *P*<0.01).

Subgroup analysis of studies based on bias risk showed that the positive association between hypothyroidism and CKD risk remained significant in studies with low bias risk and in the moderate bias risk group. There were 3 medium-quality studies and 8 high-quality studies. As shown in **Supplementary Figure 4**, the low bias risk group has high heterogeneity, but the combined effect value is stable (n=8 studies, OR = 1.69, 95% CI 1.42-2.01; *I*^2^ = 83.04%, *P*<0.01).

### Cohort study of hypothyroidism and CKD risk

Three studies analyzed the relationship between overt hypothyroidism and CKD, and one study analyzed the relationship between hypothyroidism (unable to determine whether it was overt or subclinical hypothyroidism) and CKD. These studies included a total of 4,586,856 participants, with a median follow-up time ranging from 3 to 19.6 years. Three studies had a NOS score of 8, and one study had a score of 9, indicating a low risk of bias.

**Figure 3:**
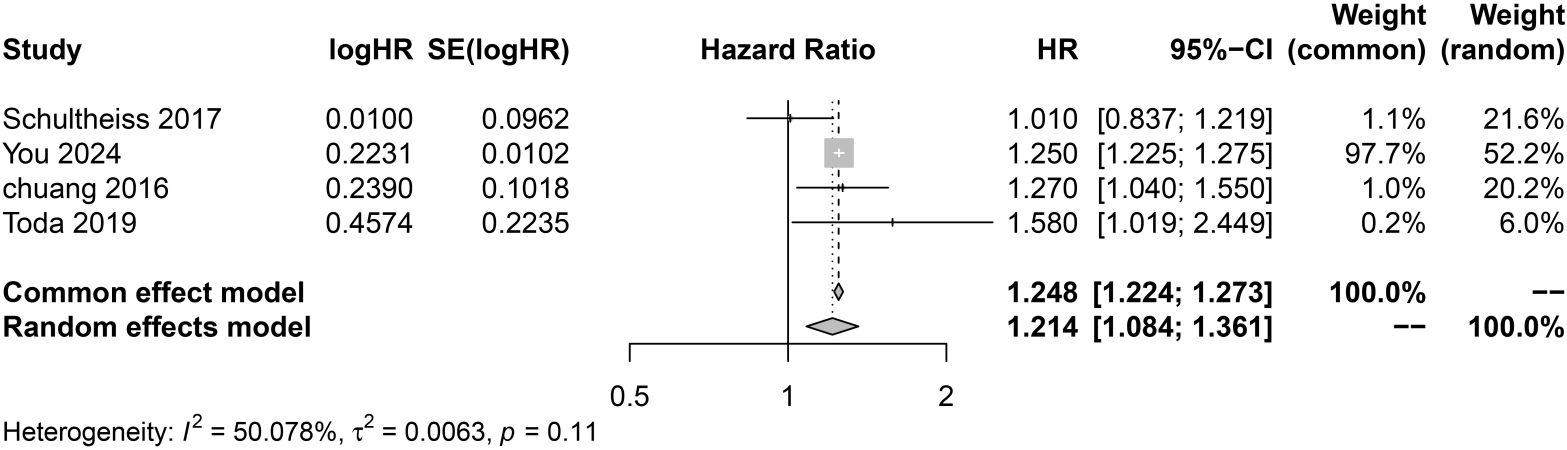
Forest plot of cohort studies on the association between hypothyroidism and CKD risk.

Due to moderate heterogeneity (*I^2^* = 50.08%), a random effects model was used for the analysis. As shown in **Figure 3**, the results of the meta-analysis of cohort studies found a significant positive correlation between hypothyroidism and the risk of CKD (HR = 1.21, 95% CI 1.08-1.36; *I*^2^ = 50.08%). This combined effect is independent of common risk factors such as age, gender, ethnicity, and hypertension.

### Sensitivity analysis

Sensitivity analyses were conducted to explore potential sources of heterogeneity between hypothyroidism and CKD risk and to validate the stability of our findings. In the exclusion analyses where one study was sequentially omitted, the overall risk estimate from cross-sectional studies did not show significant changes, with the pooled estimates of hypothyroidism and CKD risk ranging from 1.62 (95% CI: 1.39–1.93) to 1.83 (95% CI: 1.63–2.05). The sensitivity analysis results of the cross-sectional study are presented in **Supplementary Figure 5**. For cohort studies, exclusion analyses under the random-effects model indicated that the direction of pooled effect estimates remained consistent across all scenarios, with HR ranging from 1.19 (95% CI: 1.05–1.35) to 1.25 (95% CI: 1.23–1.27). In some cases, excluding individual studies led to widened confidence intervals and reduced statistical significance. This phenomenon can be explained by the small number of included cohort studies and the reduced precision after excluding influential datasets. Importantly, the direction of association remained unchanged across all analyses, supporting the robustness of the observed relationship. Results of sensitivity analyses for cohort studies are presented in **Supplementary Figure 6**.

Overall, sensitivity analyses indicate that the direction of the pooled effect is generally consistent. However, the precision of the pooled estimate is somewhat compromised due to the limited number of cohort studies and the presence of influential datasets.

### Publication bias

**Figure 4:**
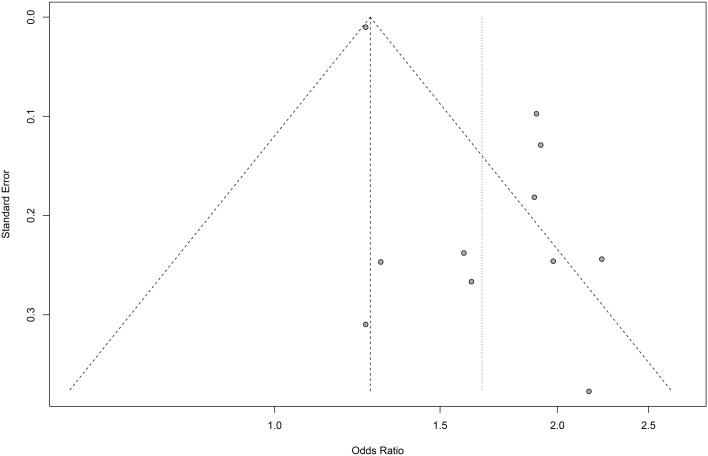
Funnel plots of publication bias.

To explore potential publication bias, funnel plots were constructed separately for cross-sectional studies and cohort studies, as shown in **Figure 4**. The funnel plot of the cross-sectional study exhibits pronounced asymmetry. Egger’s test confirmed the presence of publication bias (*P*<0.05). Trim-and-fill methods were applied under a random-effects model, interpolating six hypothetical studies. After adjustment, the pooled effect size for cross-sectional studies decreased from OR = 1.66 (95% CI: 1.43-1.94) to OR = 1.29 (95% CI: 1.06-1.57), remaining statistically significant (*P* = 0.0125). Although the association between the two remained statistically significant after adjustment, the reduced effect size suggests that publication bias may have inflated the strength of the link. Therefore, caution should be exercised when interpreting the study findings.

## Discussion

This meta-analysis included 11 cross-sectional studies and 4 cohort studies, synthesizing data from approximately 5.10 million individuals across different countries, thereby providing substantial evidence. Our findings indicate that hypothyroidism is significantly associated with an increased risk of CKD, both in cross-sectional (OR = 1.66, 95% CI 1.43–1.94) and cohort studies (HR = 1.21, 95% CI 1.08-1.36). When conducting subgroup analyses based on thyroid disease type, region, diagnostic methods for hypothyroidism, and risk of bias, the positive association between hypothyroidism and CKD risk remained consistent across different subgroups. However, some subgroups included a small number of studies and the results should be interpreted with caution. Publication bias was present in the cross-sectional studies, but the association persisted after adjustment using trim-and-fill methods, indicating the stability of the results. This large-scale, comprehensive meta-analysis provides a thorough description of the strong association between hypothyroidism and CKD.

Our research has found that hypothyroidism is positively associated with the risk of CKD. Consistent with our findings, the meta-analysis by Meuwese et al. (34) demonstrated a cross-sectional correlation between hypothyroidism and reduced eGFR levels compared with participants with normal thyroid function, although an accelerated decline in renal function was not observed in the longitudinal analysis. Furthermore, a previous meta-analysis of diabetic populations demonstrated that hypothyroidism is significantly associated with an increased risk of CKD, providing further support for the link between thyroid dysfunction and adverse renal outcomes (15). Our findings expand upon and complement the conclusions of previous meta-analyses. Wang et al. (17) reported that subclinical hypothyroidism is significantly associated with an increased risk of CKD, although the number of included studies was limited. This study integrated evidence from 15 observational studies involving approximately 5.10 million participants, significantly enhancing the statistical power and reliability of the pooled estimates. Furthermore, although the study by Wang et al. (17) primarily emphasized the impact of subclinical hypothyroidism on CKD risk, our findings indicate that both overt and subclinical hypothyroidism increase CKD risk. This association remains robust across subgroups stratified by region, diagnostic method, and risk of bias. Compared with previous studies, this research provides a more comprehensive and updated quantitative assessment of the association between hypothyroidism and CKD across diverse populations.

Evidence from Mendelian randomization (MR) studies further discusses the causal relationship between hypothyroidism and CKD. Ellervik et al. (35) and Tsao et al. (36) reported that genetically predicted elevated levels of TSH were causally associated with hyponephrosis, which supports the direction of the association observed in our analysis. In contrast, a bidirectional MR study by Xu et al. (11) suggests that chronic renal failure may have a causal effect on hypothyroidism, rather than the other way around. These findings highlight the complexity of thyroid-kidney interactions and emphasize the need for caution in interpreting causal relationships.

Hypothyroidism is positively associated with the risk of CKD, and thyroid dysfunction and CKD exhibit a bidirectional relationship, with potential mechanisms involving multiple pathways (37, 38). A cross-sectional study involving 91 CKD patients revealed a high prevalence of hypothyroidism at 30.8% among CKD patients (39). Furthermore, a significant association was observed between chronic kidney disease stages and thyroid dysfunction, supporting this bidirectional concept. Hypothyroidism adversely affects kidney structure and function through multiple pathways. Animal studies have revealed that hypothyroidism leads to a decrease in the kidney-to-body weight ratio in rats, alterations in the glomerular basement membrane, and increased permeability of glomerular capillaries (40–42). Hypothyroidism-induced renal impairment may be explained by the following mechanisms (1): reduced renin-angiotensin-aldosterone system activity leading to inadequate glomerular perfusion and water-sodium retention; (2) decreased cardiac output resulting in reduced renal blood flow; (3) elevated chronic low-grade inflammation and oxidative stress accelerating glomerulosclerosis (8, 9, 41, 43, 44). Hypothyroidism may also cause electrolyte disturbances, leading to hyponatremia and impaired urinary concentrating ability. Long-term effects may impair renal tubulointerstitial function (44–46). Recent studies have found that levothyroxine therapy for hypothyroidism can improve renal function in patients and even reverse CKD status, suggesting a close relationship between hypothyroidism and CKD (47–49). Our findings provide further evidence for the association between hypothyroidism and CKD risk, though the specific mechanisms involved warrant further investigation in future studies.

This study adds to the existing body of research by incorporating more recent evidence and updating previous findings. It not only examines the impact of subclinical hypothyroidism on CKD risk but also addresses the effects of overt hypothyroidism, thereby providing more comprehensive evidence. Second, this study incorporated research from a wider range of regions, thereby enhancing the generalizability of the findings. Finally, this study incorporated both prospective cohort studies and cross-sectional studies, thereby mitigating the effects of recall bias and selection bias while overcoming the limitation of cross-sectional studies in drawing causal inferences. Notably, the association observed in cross-sectional studies was stronger (pooled OR = 1.66), whereas the association in cohort studies was relatively weaker (pooled HR = 1.21). This discrepancy arises because cross-sectional studies reflect prevalence-based associations susceptible to reverse causality and residual confounding, whereas cohort studies provide time-to-event risk estimates that better capture temporal order between exposure and outcome. Thus, the relatively attenuated effect size in cohort studies offers more conservative and time-based evidence for the association between hypothyroidism and subsequent CKD risk.

This meta-analysis also has certain limitations. First, the small number of cohort studies included and the fact that the overall evidence comes mainly from cross-sectional studies limits the strength of causal inferences. Although cohort studies provide time-based evidence, the limited number of such studies calls for caution in interpreting the results of longitudinal studies. Second, although we collected the most fully adjusted risk estimates, each study adjusted for different confounding factors, and residual confounding from unmeasured factors cannot be entirely ruled out, which may affect the association between hypothyroidism and CKD risk. Variations in measurement standards and methods for hypothyroidism may affect the accuracy of our results. Although we analyzed the impact of different diagnostic approaches on outcomes in subgroup analyses, we cannot entirely rule out potential biases arising from measurement techniques and standards. As well, although the definition of CKD was standardized using a threshold of eGFR <60 mL/min/1.73 m². Differences in outcome determination, however, including single versus repeated eGFR measurements and the selective use of albuminuria, may be a source of residual heterogeneity that could not be fully addressed in the meta-analysis. Third, substantial heterogeneity was found in several of the pooled analyses, reflecting differences in study design, population characteristics, exposure definitions, and outcome determination. This heterogeneity may affect the precision of the pooled estimates and should be taken into account when interpreting the pooled results. Fourth, we identified publication bias in the meta-analysis of cross-sectional studies. Although bias was corrected using trim-and-fill methods and results were confirmed to be stable, caution is warranted when interpreting these findings. Small-scale negative studies may be underrepresented, and the true effect size may be smaller than initially observed. Fifth, since only English-language publications were included, relevant studies published in other languages may have been omitted, potentially introducing language bias. Furthermore, given the bidirectional relationship between thyroid dysfunction and chronic kidney disease, the possibility of reverse causality cannot be entirely ruled out. Although mechanistic studies and cohort studies provide some support for a temporal association between hypothyroidism and subsequent CKD risk, reverse causality remains impossible to completely exclude in cross-sectional studies. Finally, a large cohort study (You et al., 2024) (14) represents a large proportion of the overall sample and thus greatly affects the precision of the pooled effect estimates as well as the combined effect values, so the results of cohort studies should be interpreted with caution. Sensitivity analyses revealed that excluding this study resulted in wider confidence intervals and reduced statistical significance, yet the direction of association between hypothyroidism and CKD risk remained consistent. Given these limitations, well-designed prospective and interventional studies are necessary to further elucidate the temporal relationship and underlying mechanisms between hypothyroidism and CKD.

## Conclusion

In summary, this comprehensive meta-analysis provides substantial evidence for a significant and independent positive correlation between hypothyroidism and the risk of CKD. This association persisted and remained stable across different thyroid disease categories, geographic regions, diagnostic methods, and study quality. Given the rising prevalence and disease burden of CKD worldwide, early identification of thyroid dysfunction may have potential implications for CKD prevention, but its clinical impact remains to be clarified in future prospective studies.

## Data Availability

The raw data supporting the conclusions of this article will be made available by the authors, without undue reservation.
