# Supplementary material for: Association between hypothyroidism and risk of chronic kidney disease: evidence from a systematic review and meta-analysis
Source: Front Endocrinol (Lausanne). 2026 Feb 11;17:1704228. doi: 10.3389/fendo.2026.1704228 (PMC12932160; doi:10.3389/fendo.2026.1704228)
Supplement: Supplementary file 1 [file Table1.docx]

**Supplementary Material**

**Supplementary Table 1** The detailed search strategy for this study

| **Database** | **Search Criteria** |
| --- | --- |
| **Pubmed** | ("Hypothyroidism"[MeSH Terms] OR "Thyroid Diseases"[MeSH Terms] OR "Thyroid Gland"[MeSH Terms] OR hypothyroid*[Title/Abstract] OR "thyroid dysfunction"[Title/Abstract] OR "subclinical hypothyroidism"[Title/Abstract] OR "thyroid insufficiency"[Title/Abstract] OR "thyroid disorder*"[Title/Abstract])  AND  ("Kidney Diseases"[MeSH Terms] OR "Renal Insufficiency, Chronic"[MeSH Terms] OR "Renal Insufficiency"[MeSH Terms] OR "Kidney Failure, Chronic"[MeSH Terms] OR "Chronic Kidney Disease"[Title/Abstract] OR CKD[Title/Abstract] OR "chronic renal insufficiency"[Title/Abstract] OR "renal dysfunction"[Title/Abstract] OR "kidney insufficiency"[Title/Abstract])  AND  ("Epidemiologic Studies"[MeSH Terms] OR "Cohort Studies"[MeSH Terms] OR "Case-Control Studies"[MeSH Terms] OR "Cross-Sectional Studies"[MeSH Terms] OR epidemiolog*[Title/Abstract] OR cohort[Title/Abstract] OR case-control[Title/Abstract] OR "cross-sectional"[Title/Abstract] OR prospective[Title/Abstract] OR observational[Title/Abstract])  Filters: Humans |
| **Embase** | ('hypothyroidism'/exp OR 'thyroid disease'/exp OR hypothyroid*:ab,ti OR 'thyroid dysfunction':ab,ti OR 'subclinical hypothyroidism':ab,ti OR 'thyroid insufficiency':ab,ti OR 'thyroid disorder*':ab,ti)  AND  ('chronic kidney disease'/exp OR 'renal insufficiency'/exp OR 'kidney failure'/exp OR 'chronic renal insufficiency':ab,ti OR 'CKD':ab,ti OR 'renal dysfunction':ab,ti OR 'kidney insufficiency':ab,ti)  AND  ('epidemiology'/exp OR 'cohort analysis'/exp OR 'case control study'/exp OR 'cross-sectional study'/exp OR epidemiolog*:ab,ti OR cohort:ab,ti OR case-control:ab,ti OR 'cross-sectional':ab,ti OR prospective:ab,ti OR observational:ab,ti)  Filters: Humans |
| **Cochrane Library** | (hypothyroidism OR "thyroid dysfunction" OR "subclinical hypothyroidism" OR "thyroid insufficiency" OR "thyroid disorder*")  AND  ("chronic kidney disease" OR CKD OR "renal insufficiency" OR "chronic renal insufficiency" OR "renal dysfunction" OR "kidney insufficiency")  AND  (epidemiology OR cohort OR "case-control" OR "cross-sectional" OR observational OR prospective) |
| **Web of Science** | TS=(hypothyroidism OR "thyroid dysfunction" OR "subclinical hypothyroidism" OR "thyroid insufficiency" OR "thyroid disorder*")  AND  TS=("chronic kidney disease" OR CKD OR "renal insufficiency" OR "chronic renal insufficiency" OR "renal dysfunction" OR "kidney insufficiency")  AND  TS=(epidemiology OR cohort OR "case-control" OR "cross-sectional" OR observational OR prospective)  Refined by: Document Types=(Article) AND Languages=(English) |

The database search was performed in four electronic databases: PubMed (via Medline), Embase, Cochrane Library, and Web of Science, from inception to September 1, 2025. Since all databases require different syntaxes, specific search strategies were developed for each.

**Supplementary Table 2.** Risk of bias assessment (Agency for Healthcare Research and Quality).

| Study | 1)Define the source of information (survey, record review) | 2)List inclusion and exclusion criteria for exposed and unexposed subjects (cases and controls) or refer to previous publications | 3)Indicate time period used for identifying patients | 4)Indicate whether or not subjects were consecutive if not population-based | 5)Indicate if evaluators of subjective components of study were masked to other aspects of the status of the participants | 6)Describe any assessments undertaken for quality assurance purposes (e.g, test/retest of primary outcome measurements) | 7)Explain any patient exclusions from analysis | 8)Describe how confounding was assessed and/or controlled | 9)If applicable, explain how missing data were handled in the analysis | 10)Summarize patient response rates and completeness of data collection | 11)Clarify what follow-up, if any, was expected and the percentage of patients for which incomplete data or follow-up was obtained | Quality  score |
| --- | --- | --- | --- | --- | --- | --- | --- | --- | --- | --- | --- | --- |
| Huang(2020） | Yes | Yes | Yes | Yes | No | Unclear | Yes | Yes | Unclear | Yes | Unclear | 8 |
| Shimizu(2022) | Yes | Yes | Yes | Yes | No | Unclear | Yes | Yes | Unclear | Unclear | Unclear | 7 |
| Jia(2015） | Yes | Yes | Yes | Yes | No | Unclear | Yes | Yes | Unclear | Unclear | Unclear | 7 |
| Gopinath(2013) | Yes | Yes | Yes | Yes | No | Yes | Yes | Yes | Unclear | Yes | Unclear | 9 |
| Kim(2023) | Yes | Yes | Yes | Yes | No | Unclear | Yes | Yes | Unclear | Yes | Unclear | 8 |
| Schultheiss(2017) | Yes | Yes | Yes | Yes | No | Yes | Yes | Yes | Unclear | Yes | No | 9 |
| Johnson(2020) | Yes | Yes | Yes | Unclear | No | Unclear | Yes | Unclear | No | Unclear | Unclear | 5 |
| Peixoto De Miranda(2017) | Yes | Yes | Yes | Yes | No | Yes | Yes | Yes | Unclear | Yes | Unclear | 9 |
| Åsvold(2011) | Yes | Yes | Yes | Yes | No | Yes | Yes | Yes | Unclear | Yes | Unclear | 9 |
| Toda(2019) | Yes | Yes | Yes | Yes | No | Unclear | Yes | Yes | Unclear | Yes | Unclear | 8 |
| Chang(2018) | Yes | Yes | Yes | Yes | No | Unclear | Yes | Yes | Unclear | Yes | Unclear | 8 |

Y, Yes; N, No; U, Unclear; an item would be scored ‘0’ if it was answered ‘NO’ or ‘UNCLEAR’; if it was answered ‘YES’, then the item scored ‘1’ (Question 5 take reverse scoring).

**Supplementary Table 3.** Risk of bias assessment (Newcastle-Ottawa Quality Assessment Scale criteria).

| Study | Selection | | | | Comparability | Outcome | | | Quality  score |
| --- | --- | --- | --- | --- | --- | --- | --- | --- | --- |
|  | Representativeness of the exposed cohort | Selection of the non-exposed cohort | Ascertainment of exposure | Demonstration that the outcome of interest was not present at start of the study | Comparability of cohorts on the basis of the design or the analysis | Ascertainment of outcome | Was follow-up long enough for outcomes to occur? | Adequacy of follow-up of cohorts |  |
| Schultheiss(2017) | * | * | * | * | ** | * | * | * | 9 |
| You, primary cohort(2024) | * | * | * | * | ** | * | / | * | 8 |
| chuang(2016) | * | * | * | * | ** | * | / | * | 8 |
| Toda(2019) | * | * | * | * | ** | * | / | * | 8 |

**Supplementary Figure 1** Forest plot for subgroup analysis by type of thyroid disease


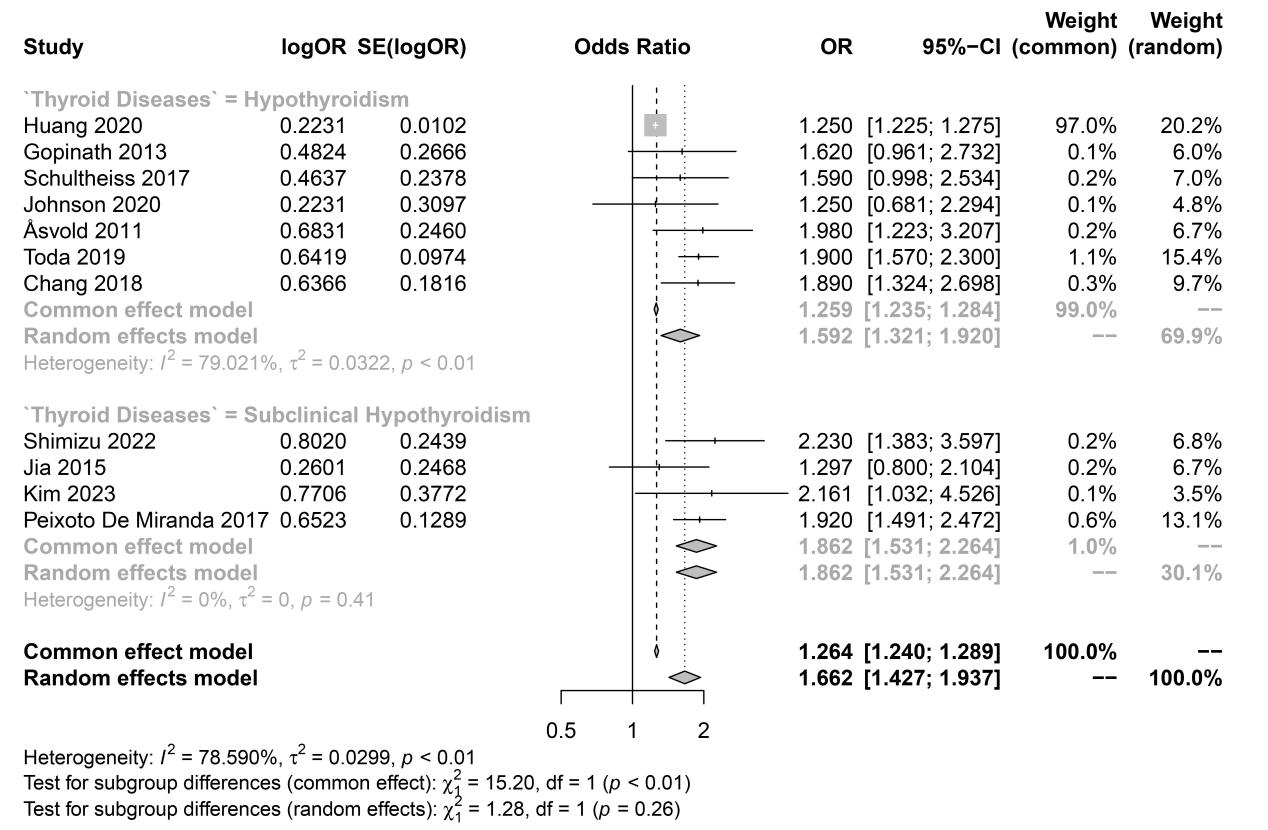


**Supplementary Figure 2** Forest plot for subgroup analysis by region

**
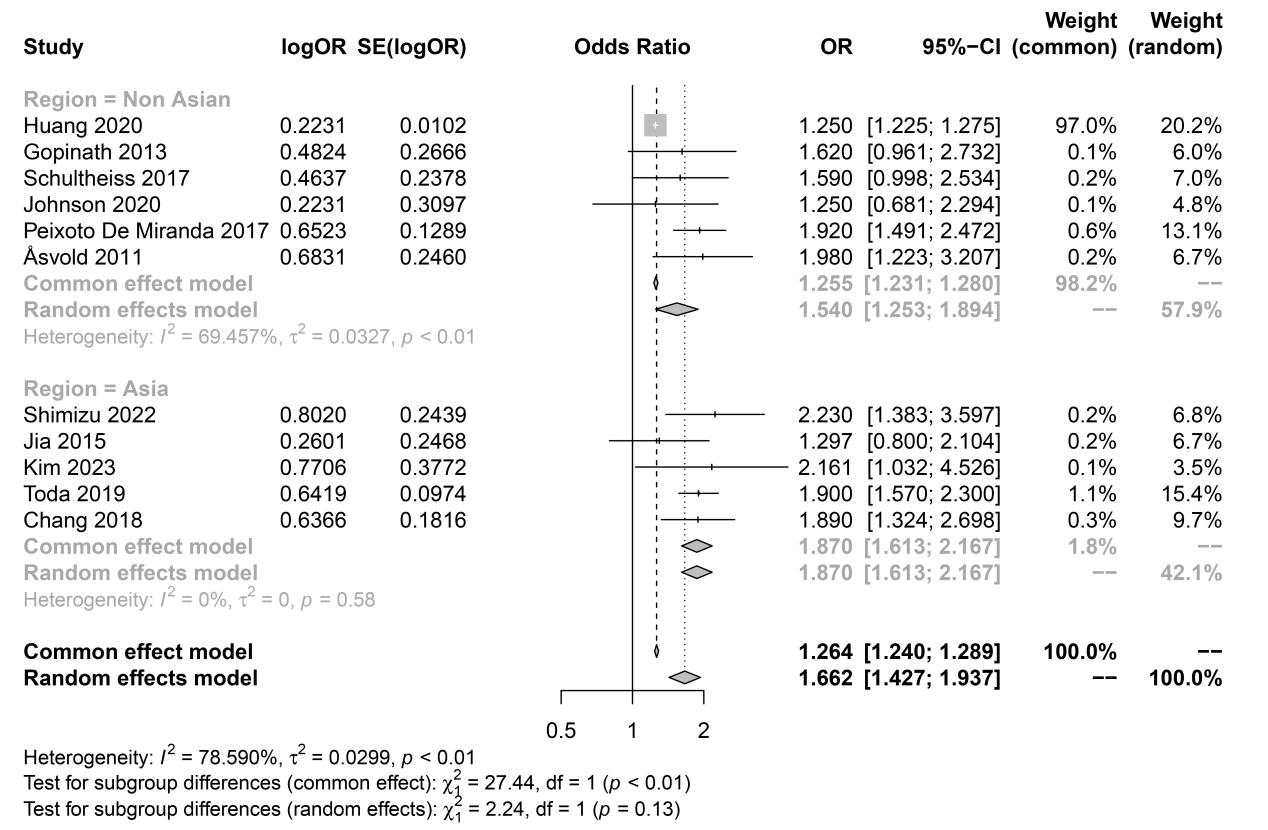
**

**Supplementary Figure 3** Forest plot for subgroup analysis by diagnostic method

**
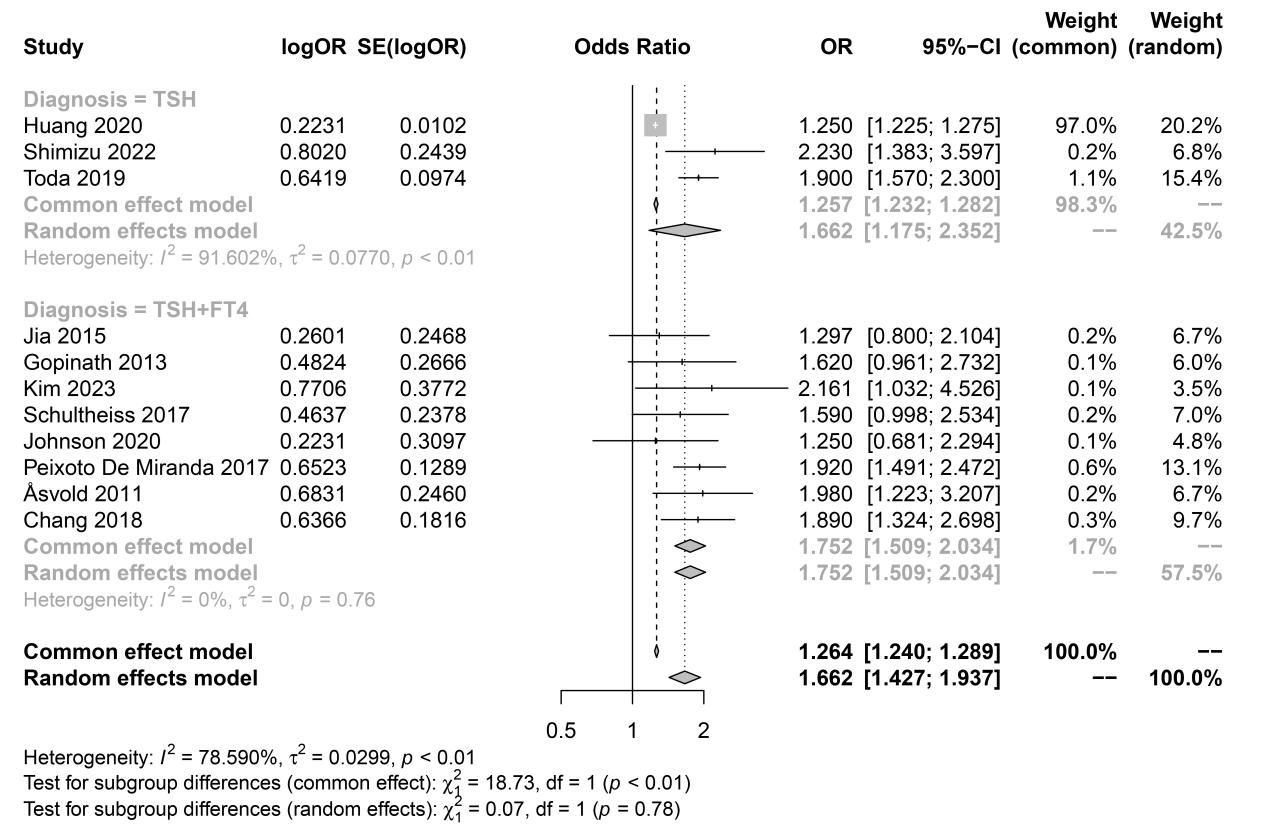
**

**Supplementary Figure 4** Forest plot for subgroup analysis by the quality of research


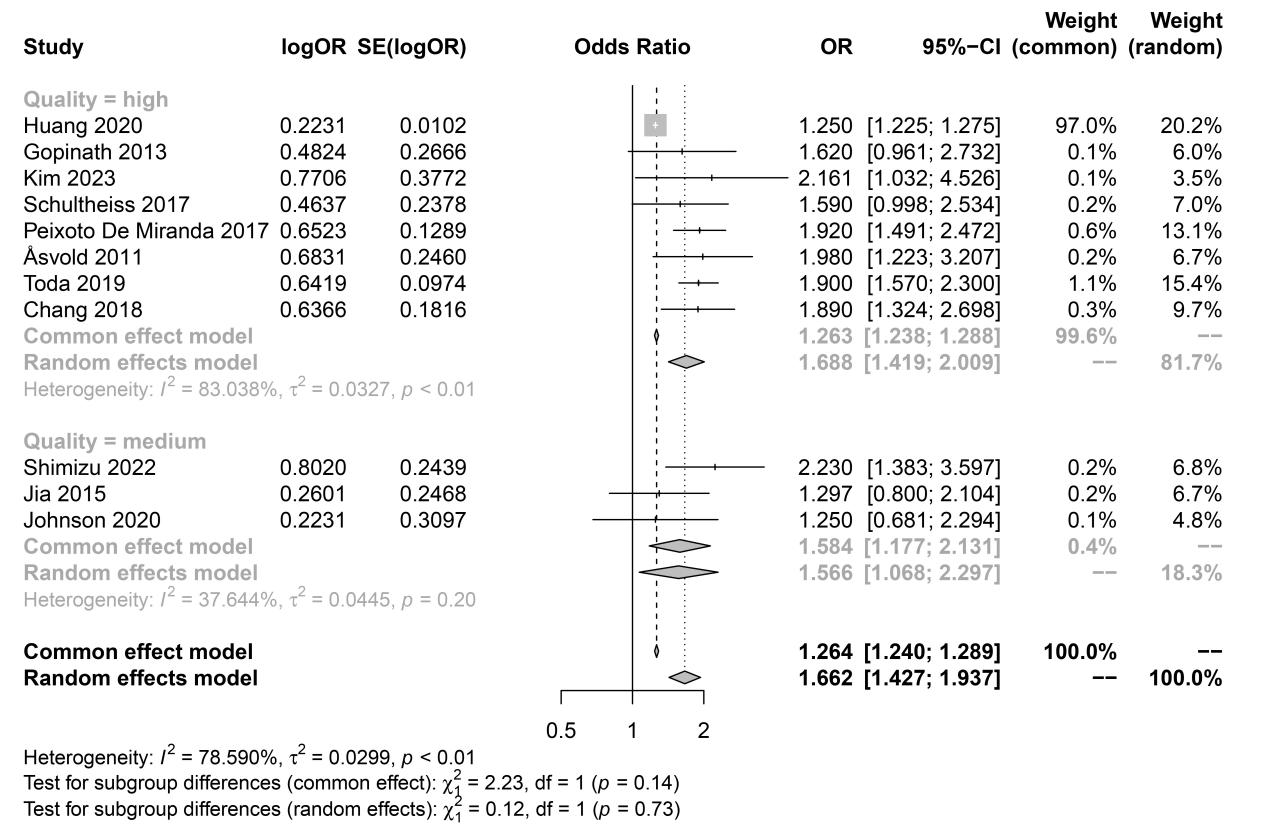


**Supplementary Figure 5** Sensitivity analysis of cross-sectional studies

**
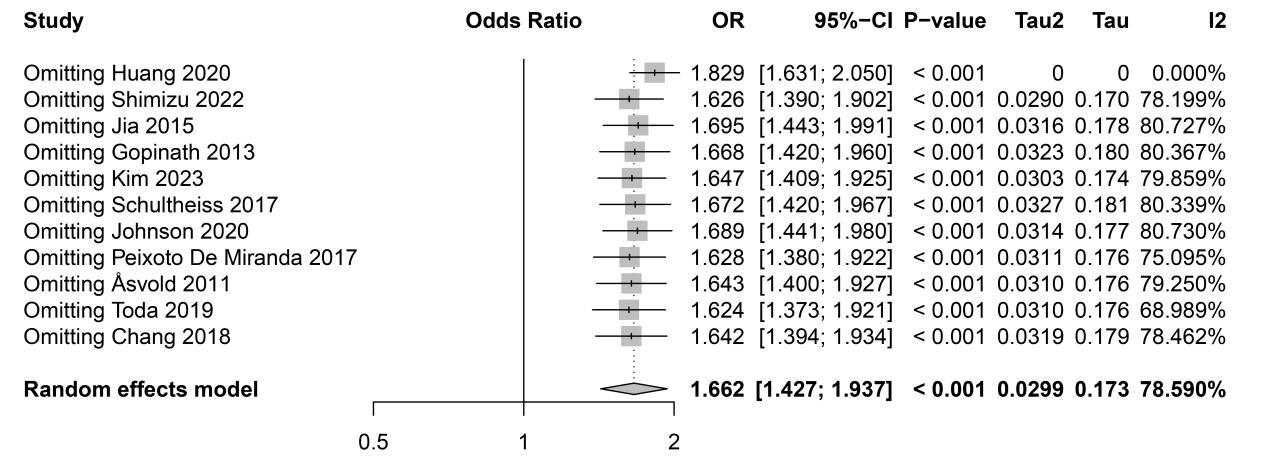
**

**Supplementary Figure 6** Sensitivity analysis of cohort studies

**
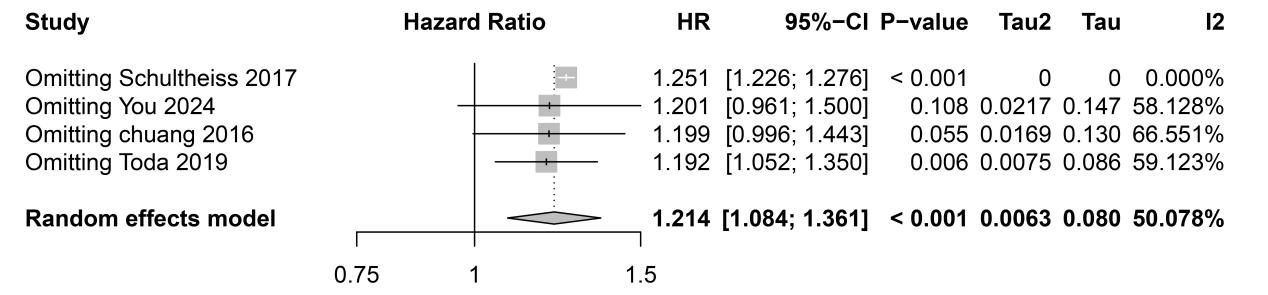
**
